# Bayesian correction model for over-estimation and under-estimation of liver cancer incidence in Iranian neighboring provinces 

**Published:** 2017

**Authors:** Nastaran Hajizadeh, Ahmad Reza Baghestani, Mohamad Amin Pourhoseingholi, Hadis Najafimehr, Zeinab Fazeli, Luca Bosani

**Affiliations:** 1 *Basic and Molecular Epidemiology of Gastrointestinal Disorders Research Center, Research Institute for Gastroenterology and Liver Diseases, Shahid Beheshti University of Medical Sciences, Tehran, Iran.*; 2 *Department of Biostatistics, Faculty of Paramedical Sciences, Shahid Beheshti University of Medical Sciences, Tehran, Iran.*; 3 *Gastroenterology and Liver Diseases Research Center, Research Institute for Gastroenterology and Liver Diseases, Shahid Beheshti University of Medical Sciences, Tehran, Iran.*; 4 *Foodborne and Waterborne Diseases Research Center, Research Institute for Gastroenterology and Liver Diseases, Shahid Beheshti University of Medical Sciences, Tehran, Iran*; 5 *Department of Infectious Diseases, Istituto Superiore di Sanità, Roma, Italy *

**Keywords:** Liver cancer, incidence registries, misclassification, Bayesian method, Iran

## Abstract

**Aim:**

The aim of this study was to obtain more accurate estimates of the liver cancer incidence rate after correcting for misclassification error in cancer registry across Iranian provinces.

**Background:**

Nowadays having a thorough knowledge of geographic distribution of disease incidence has become essential for identifying the influential factors on cancer incidence.

**Methods:**

Data of liver cancer incidence was extracted from Iranian annual of national cancer registration report 2008. Expected coverage of cancer cases for each province was calculated. Patients of each province that had covered fewer cancer cases than 100% of its expectation, were supposed to be registered at an adjacent province which had observed more cancer cases than 100% of its expected coverage. For estimating the rate of misclassification in registering cancer incidence, a Bayesian method was implemented. Beta distribution was considered for misclassified parameter since its expectation converges to the misclassification rate. Parameters of beta distribution were selected based on the expected coverage of cancer cases in each province. After obtaining the misclassification rate, the incidence rates were re-estimated.

**Results:**

There was misclassification error in registering new cancer cases across the provinces of Iran. Provinces with more medical facilities such as Tehran which is the capital of the country, Mazandaran in north of the Iran, East Azerbaijan in north-west, Razavi Khorasan in north-east, Isfahan in central part, and Fars and Khozestan in south of Iran had significantly higher rates of liver cancer than their neighboring provinces. On the other hand, their neighboring provinces with low medical facilities such as Ardebil, West Azerbaijan, Golestan, South and north Khorasans, Qazvin, Markazi, Arak, Sistan & balouchestan, Kigilouye & boyerahmad, Bushehr, Ilam and Hormozgan, had observed fewer cancer cases than their expectation.

**Conclusion:**

Accounting and correcting the regional misclassification are necessary for identifying high risk areas of the country and effective policy making to cope with cancer.

## Introduction

 Liver cancer is the 5th most common cause of cancer incidence and the second most common cause of cancer death in the world (1). It is estimated to be responsible for nearly 782,000 of new cases of cancer and 746,000 deaths based on Globocan report 2012 (2). Hepatocellular carcinoma (HCC) represents the major histologic type of liver cancer and accounts for 70 to 85 percent of the cases. Mortality to incidence ratio of HCC is 0.95, thus the geographical patterns of mortality and incidence of this cancer are similar (2, 3). More than 80 percent of new cases occurs in less developed countries that about half of them belongs to China alone (2). The major risk factors for liver cancer, are Hepatitis C Virus (HCV) and Hepatitis B Virus (HBV) infections (3) . HBV is the most common cause of liver cancer in Iran (4, 5). It is estimated that 1.5 million people in the country are infected with this type of virus and 15% to 40% of them are at risk of developing liver cancer or cirrhosis (6, 7). The other known risk factors for liver cancer are gender (it is more common in males than in females), race (Pacific Islanders and Asian Americans have the highest rates of incidence and Whites have the lowest rates), aflatoxins and tobacco use, cirrhosis, non-alcoholic fatty liver disease, obesity and heavy alcohol use (8). The high incidence regions are Eastern and South-Eastern Asia, the intermediate incidence rates regions are Southern Europe and Northern America, and the regions of lowest rates are Northern Europe and South-Central Asia (2). Iran is located in Middle East, an area with low risk of developing liver cancer (1, 9) and an annual incidence rate that is much less than 5 per 100,000 populations (10). but since prognosis for liver cancer is very poor, the true prevalence of liver cancer is unknown in the country, so it is not considered as an uncommon malignancy (2, 3).

Nowadays having a true knowledge of geographic distribution of disease incidence has become essential for identifying the influencing factors on cancer incidence and planning for cancer control and prevention (11, 12). 

Cancer registries are known as the main resources for data of mortality, incidence, prevalence and survival for different disease which are recorded in a systematic manner. Registered data is the basis for health policy makers for planning for cancer control and prevention, evaluation of interventions and cancer screening programs, and allocating available resources to the provinces based on their needs to healthcare facilities.

Confronting a cluster which has a significantly high incidence rate, this question comes to mind that what would be the underlying causal mechanism? Of course, -investigators first, focus on the risk factors of the disease (13). But major differences in incidence rate of liver cancer in adjacent provinces that are very similar in exposure with risk factors are only justifiable with existence of misclassification error in registration system. Misclassification error is the disagreement between the observed and the true value. This error occurs because some people prefer to get diagnostic and treatment services in a neighboring province that is more equipped and some of that patients are registered in the province who have been referred for treatment without reporting their own address in their permanently living province. The expected coverage of cancer cases in different provinces of the country is the indicator of existence of misclassification error in registry system; forasmuch as the observed rate of cancer incidence is more than the expected rate in some provinces, and on the other hand, it is much less than expected rate in their neighboring provinces (14). It happens while it is expected that the rate of cancer incidence be about the same in neighboring provinces due to similarity in lifestyle and environmental exposures.

Misclassification error in cancer registry makes the registry systems inaccurate for estimating the risk of cancers in different places. Consequently, resource allocation and planning for preventive and therapeutic interventions are affected and most probably would be wrong (15, 16).

For correcting the misclassification error, two approaches can be used; the first is reviewing medical records and validating a small sample of data to gain an estimate of misclassification rate, and then extending its results to the population under study to correct for misclassification (17). This method is known as one of the costly and time consuming approaches and in some cases it is not feasible because it may not be possible to obtain a valid sample. The second approach which is faster and more cost effective and is not in need of data validation, is using Bayesian method for estimating the rate of misclassification error. This method is a statistical method that makes the possibility of combining expert’s prior knowledge about misclassification rate with the observed data to obtain a posterior distribution which will be the basis of inferences (18, 19). Also unobserved variables such as individual’s true information in the presence of misclassification error, can be accommodated by using the Bayesian method (4, 16).

The aim of this study is to estimate the rate of misclassification error in registering cancer incidence in adjacent provinces by implementing Bayesian method and re-estimating the rate of liver cancer in each province after correcting misclassification. 

## Methods

Registered incidence data due to liver cancer in 2008 were extracted from the National Cancer Registry (NCR) of Iran that is publishes annually (14). The data of year 2008 was the last published report. NCR collects its data in cooperation with medical universities of the country. New cancer cases which confirmed by pathology centers or other diagnostic centers are recorded by medical universities of the country. Recorded cases are entered to software that is designed by ministry of health. Ministry of health sends the data back to medical universities of each province after coding the cancers based on 10^th^ revision of international coding of disease (ICD10) and removing duplicates. In this way, the observed number of cancer cases in each medical university is obtained. The expected coverage of cancer cases is also calculated for each university. It is considered to be 113 per 100,000 population that are covered by each medical university. The observed number of cancer cases was divided to the expected number of cancer cases and the output was multiplied in 100; in this way the percent of expected coverage was calculated for each university.

The data for the province that had an expected coverage less than 100% were entered to the Bayesian model in the form of vector y_1_=[y_11_,y_21_,…,y_r1_]ʹ that contains the age standardized rate of liver cancer for male and female in 4 age groups (0-14 years old, 15-49 years old, 50-69 years old and over than 70 years old); and vector y_2_=[y_12_,y_22_,…,y_r2_]ʹ was used for the same information for a neighboring province with more than 100% expected coverage which includes some of the patients that were actually belonged to the first group. The r subscript denotes the covariate patterns that are made by combinations of age-sex groups.

Since vectors y_1_ and y_2 _contain count data, a Poison distribution was considered for them (20). An informative Beta distribution was assumed as prior information for θ parameter which is denoted the probability of registering cases in misclassified group (21).

The expected coverage of cancer cases in each province were selected as prior values for the parameters of Beta distribution. In this way, the expectation formula of Beta distribution, tends to the misclassified rate. Since misclassified parameter (θ) is unknown; then a latent variable approach was employed for correcting the misclassification error (16, 20). Latent variable was considered as the number of cases that are incorrectly registered in the misclassified group and should be returned to their own group. A Binomial distribution was assigned to the latent variable. By multiplying the prior distribution in likelihood, the posterior distribution was obtained. Finally, a sample was produced from the posterior distribution by using a Gibbs sampling algorithm (22, 23). Then the produced samples were averaged and reported as misclassification rate. At last, the rates of liver cancer incidence were re-estimated for each province after correcting for misclassification error. Analyses were performed using R software, version 3.2.0. 

## Results

Among 30 provinces of Iran, 21 provinces were selected for correcting the misclassification error in registering the incidence of liver cancer between adjacent provinces. This selection was according to their expected coverage of cancer cases. In the other nine provinces, cancer rates were remained unchanged since the observed cases of cancer were about the same as the expected rate.

The Age standardized rate (ASR) of liver cancer for female was 1.56 per 100,000 population which is equivalent to 376 cases and was 2.03 per 100,000 population for male which is equivalent to 574 cases.

**Graph 1:**
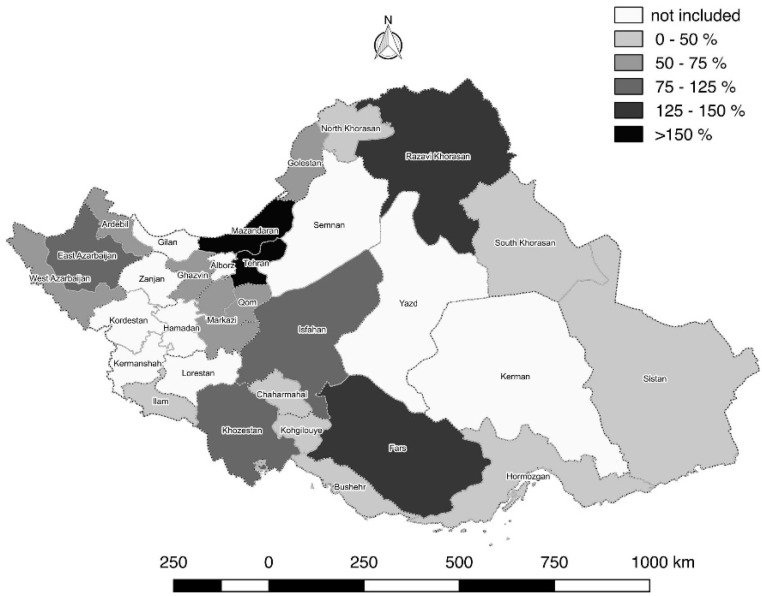
The expected coverage of cancer cases in provinces of Iran according to cancer registry report (2008)

Tehran province (capital of Iran) which is an equipped province in central part of Iran was observed 155.63% of its expected number of new cancer cases in 2008; whereas its adjacent provinces, such as Qom, Markazi and Qazvin provinces have just covered 53.9%, 69.6% and 66.3% of their expected coverage, respectively. It is clearly indicated the existence of misclassification in registering cancer incidence from deprived provinces in full featured provinces. The calculated expected coverage for the other provinces is also reported for year 2008 (Table 1 and Graph 1). 

**Table 1:** Expected coverage of cancer cases in provinces of Iran (2008)

Province	Expected Coverage
South khorasan	41.40
Razavi khorasan	143.74
Tehran	155.63
Markazi	69.60
Sistan	18.44
Qom	53.90
Ghazvin	66.30
Khozestan	101.19
Ilam	39.40
Bushehr	25.00
Golestan	50.80
Mazandaran	338.45
North khorasan	34.80
Chaharmahal	37.00
Isfahan	106.98
Kohgilouye	25.10
Hormozgan	19.00
Fars	127.65
Ardebil	63.00
East azarbaijan	123.60
West azarbaijan	69.00

**Table 2:** Age standardized rate of liver cancer before and after Bayesian correction in provinces of Iran (2008)

Province	Before Bayesian Correction			After Bayesian Correction		
	Female	Male	Total	Female	Male	Total
South khorasan	0.49	0.44	0.47	1.18	1.06	1.12
Razavi khorasan	0.95	2.19	1.57	0.39	0.89	0.64
Tehran	2.06	2.4	2.23	1.96	2.28	2.12
Markazi	0.4	0.24	0.32	0.82	0.49	0.66
Sistan	0.47	0.78	0.63	1.43	2.37	1.90
Qom	0.46	0.49	0.48	1.01	1.08	1.05
Ghazvin	0.27	0.36	0.32	0.57	0.76	0.67
Khozestan	4.53	5.65	5.09	3.98	4.96	4.47
Ilam	0.65	0.91	0.78	1.85	2.60	2.23
Bushehr	1.27	0.36	0.82	4.93	1.40	3.16
Golestan	0.44	0.87	0.66	0.77	1.52	1.14
Mazandaran	0.65	1.43	1.04	0.50	1.10	0.80
North khorasan	0.82	0.89	0.86	1.81	1.96	1.89
Chaharmahal	0.98	1.04	1.01	1.57	1.67	1.62
Isfahan	0.71	0.94	0.83	0.45	0.59	0.52
Kohgilouye	1.08	1.99	1.54	1.77	3.26	2.51
Hormozgan	0.2	0.82	0.51	0.87	3.58	2.23
Fars	2.09	2.33	2.21	1.48	1.65	1.56
Ardebil	1.16	3.23	2.20	1.40	3.90	2.65
East azarbaijan	0.84	1.1	0.97	0.53	0.69	0.61
West azarbaijan	0.15	0.9	0.53	0.24	1.45	0.84

**Graph 2.**
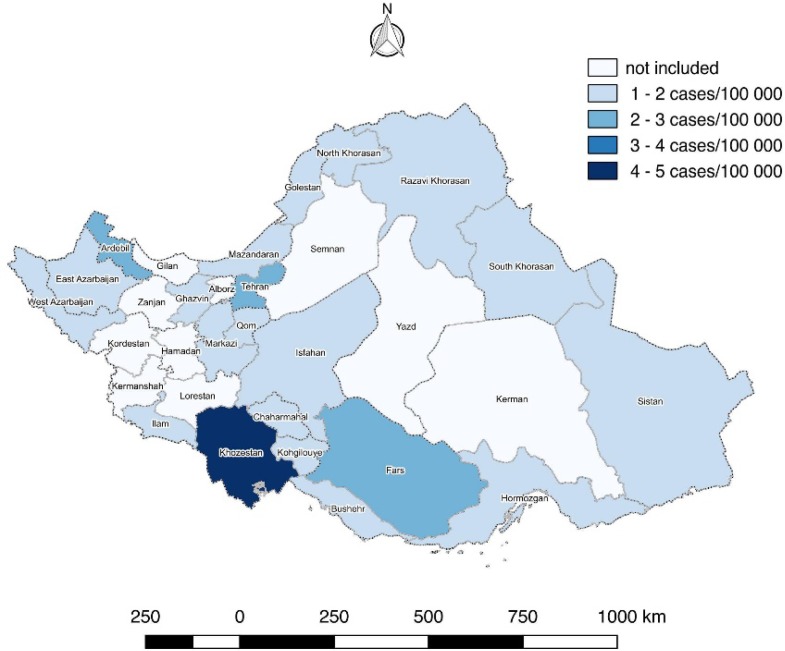
Age standardized rate (ASR) of liver cancer before Bayesian correction in provinces of Iran (2008)

**Graph 3:**
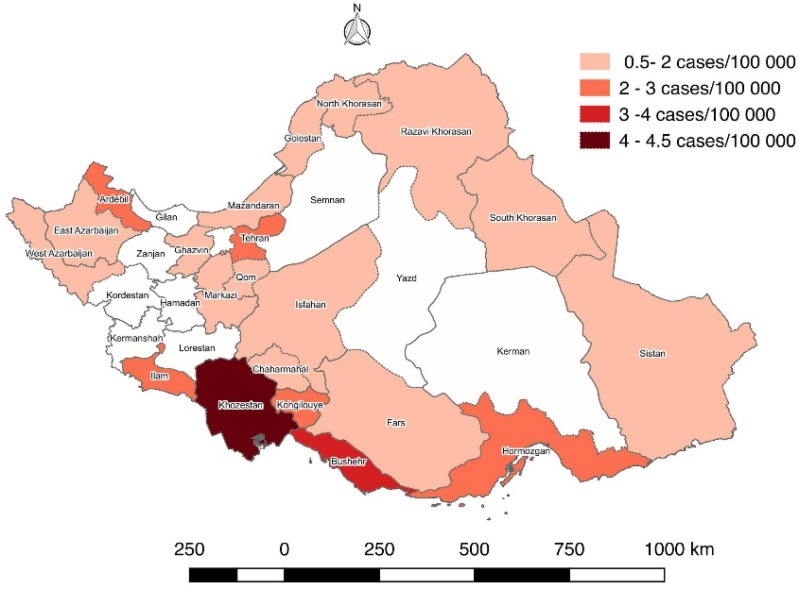
Age standardized rate (ASR) of liver cancer after Bayesian correction in provinces of Iran (2008)

The outputs of Bayesian model suggest that there was 65% misclassification in registering liver cancer incidence from Qom in Tehran province, 73% misclassification from Markazi province in Tehran, 74% misclassification from Qazvin province in Tehran, 23% misclassification from Chaharmahal & bakhtyari province in Isfahan which is one of the best equipped provinces of the country, 16% misclassification from Kohgilouye & boyerahmad province in Isfahan province, 38% misclassification from Golestan province in Mazandaran province, in the north of Iran, 72% misclassification from Bushehr province in Khozestan province, in the south part of the country that has more health facilities in comparison with its neighboring provinces, 73% misclassification from Ilam province in Khozestan, 64% misclassification from Hormozgan province in Fars province which is one of the few provinces in the southern half of the country which has equipped healthcare facilities, 13% misclassification from Ardebil province in East Azerbaijan which is the facilitate neighboring province of Ardebil in north west of Iran, 42% misclassification from West Azerbaijan province in East Azerbaijan province, 42% from North Khorasan in Razavi Khorasan province, 58% misclassification from South Khorasan in Razavi Khorasan province, and 51% misclassification from Sistan & balouchestan province located in south-west of Iran in Razavi Khorasan.

ASR of liver cancer before and after Bayesian correction are reported for each province in Table 2 and corresponding Graphs which indicating the difference before and after Bayesian correction (Graph 2 and Graph 3).

## Discussion

There was a remarkable misclassification in cancer registry system in Iran. The estimated rates of misclassification were more than 50% for Qazvin, Ilam, Markazi, Bushehr, Qom, Hormozgan, South Khorasan, and Sistan, which all of them are accounted as the most deprived provinces of the country. So the true rate of liver cancer was more than the registered rate in those provinces. Thus in addition to the importance of accurate recording of data, more medical facilities should be allocated to the deprived provinces. With equipping all the provinces, people do not need to go to other provinces for diagnosis and treatment of their disease.

The findings of a study on incidence of liver cancer in Iran, showed that the incidence rate of this cancer is increasing in the country, especially in males and higher age groups (1). The incidence of this cancer is also increasing in many countries such as the Central America, United States and Europe (6). Thorough information does not exist about the exact rate of liver cancer in Iran. Based on the results of a study, some provinces such as Tehran as the capital of Iran, Guilan, Kerman, Fars and Razavi Khorasan, have a low but significantly higher incidence rates in comparison with their neighboring provinces (24). All the mentioned provinces are accounted as full featured provinces of Iran. The results of this study are also confirming the existence of misclassification error between adjacent provinces.

Usually spatial analysis is performed to obtain information about the geographical spread of cancers for identifying high risk areas to carry out preventive measures (25, 26). But in most of those analysis cancer registry data is used regardless of existed misclassification error in registering cancer incidence. As a result, the risk of cancer is overestimated in one area and underestimated in another one. It consequently, affects prioritizations for allocating healthcare facilities. By implementing the described Bayesian method, existence of misclassification error is taken into account and more accurate estimates of cancer rates are provided. If corrected data be used in spatial analysis, more accurate results will be provided about the spatial pattern of disease.

In conclusion there is some regional misclassification error in cancer registry system. Regionally misclassified data leads to underestimation of cancer rate in some provinces. So if the misclassified registered data be the basis of decisions of policy makers, some provinces will always remain deprived; because this misconception arises that people in those area are less likely to develop cancer.

In most Asian countries, early detection services are limited. In Iran, as in other Middle East countries, majority of liver cancer cases are diagnosed in intermediate or advanced stages of the disease (27). Many people do not have affordability for screening tests or medical treatments. For reducing the incidence and mortality of cancers, it is necessary to recognize these groups of people (4). Vaccination, using screening tests, changes in treatment and prevention strategies, and awareness about symptoms or risk factors of liver cancer can make a substantial change in the future burden of disease (28). Planning national and sub-national programs to improve the quality and accuracy of cancer registry system and correcting its errors are also necessary for cancer control.

In the absence of valid data, Bayesian approach is a fast and cost effective method to correct for misclassification error in cancer incidence registry data.
